# Dissecting the Structural Features of Folding Asymmetry and Transient Misfolding in a Multidomain PDZ Scaffold

**DOI:** 10.1002/smsc.202500511

**Published:** 2025-12-10

**Authors:** Valeria Pennacchietti, Mariana Di Felice, Julian Toso, Lucia Marcocci, Paola Pietrangeli, Eduarda S. Ventura, Francesca Malagrinò, Angelo Toto, Stefano Gianni

**Affiliations:** ^1^ Dipartimento di Scienze Biochimiche “A. Rossi Fanelli” Sapienza Università di Roma P.le Aldo Moro 5 00185 Rome Italy; ^2^ Laboratory affiliated to Istituto Pasteur Italia Fondazione Cenci Bolognetti 00161 Rome Italy; ^3^ Dipartimento di Medicina clinica, sanità pubblica, scienze della vita e dell'ambiente Università dell’Aquila Piazzale Salvatore Tommasi 1 67010 L'Aquila – Coppito Italy

**Keywords:** kinetics, multidomain proteins, protein folding, site‐directed mutagenesis

## Abstract

Understanding how multidomain proteins fold, avoid misfolding, and maintain functional regulation represents a critical problem in structural biology, with broad relevance for biotechnology. In this study, the tandem PDZ1–PDZ2 domains of the scaffold protein X11, a system of particular interest due to its previously reported asymmetric folding and unfolding behavior as well as its propensity to form transient misfolded intermediates, are investigated. Through extensive mutational work and in‐depth kinetic folding analysis, the folding behavior of this multidomain construct is dissected, and a comparative analysis with its isolated PDZ domains is also performed. The results reveal that folding and unfolding proceed through distinct pathways with PDZ2 folding rapidly and independently, while PDZ1 folds more slowly and only upon engagement of an autoinhibitory regulatory tail. Despite these differences, the folding mechanisms of each domain are conserved when studied in isolation, with deviations largely confined to functionally relevant and frustrated regions. The results also allow to depict the structural features of a misfolded intermediate that competes with productive folding and is stabilized by non‐native interdomain contacts. Strikingly, this misfolded trap retains elements of the PDZ2 folding nucleus, an unexpected finding that allows us to draw broader conclusions about how transient misfolding can arise even from native‐like structural motifs. We discuss these results in light of prior studies on multidomain proteins.

## Graphical abstract

This study reveals how a multidomain protein can misfold through subtle internal frustrations rather than by swapping entire domains. Using Φ‐value analysis, the authors map the structural features that govern folding and misfolding in the X11 PDZ1–PDZ2 scaffold. Their findings provide rare insight into how domain interactions and denatured‐state constraints shape the complex folding behavior of large proteins.

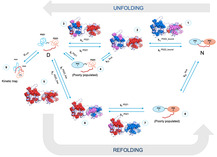

## 1 Introduction

The study of folding of multidomain proteins is of special interest in nanoscience. In fact, it exemplifies how complex, functional architectures can emerge through self‐organization at the molecular level. The intrinsic ability of proteins to self‐assemble into precise and active conformations makes them particularly attractive both as models and as components in synthetic nanosystems.^[^ ^1^, ^2^, ^3^, ^4^ ^]^ In proteins, interactions between domains often go beyond simple modularity, involving supramodular chemistry—cooperative effects between structural units that give rise to emergent behaviors not predictable from the properties of the individual parts.^[^ ^5^, ^6^, ^7^, ^8^ ^]^

Although multidomain proteins represent roughly 75% of the human proteome,^[^ ^9^ ^]^ our current understanding of protein folding is predominantly based on single‐domain systems.^[^ ^10^ ^]^ This bias largely originates from the experimental complexity of studying larger, more intricate proteins, which are often particularly elusive to quantitative analyses. Furthermore, the relative ease of expressing and characterizing isolated domains has further reinforced the widespread, yet reductive, assumption that individual domains fold and operate independently. Nevertheless, transient interactions between domains can introduce complex folding behaviors, which demand a careful investigation. For example, multidomain proteins may display interdomain interactions, tuning stability and/or folding cooperativity, as well as competing misfolding events.^[^ ^11^, ^12^, ^13^, ^14^, ^15^, ^16^, ^17^, ^18^ ^]^ These features expand the conformational landscape and often obscure the folding mechanism, making multidomain proteins much harder to characterize. As a result, despite their biological prevalence and functional importance, our understanding of how multidomain folding remains relatively limited.

Within this framework, tandem repeats of PDZ domains offer a compelling model system to explore how modularity and cooperativity contribute to protein folding.^[^ ^16^, ^17^, ^18^, ^19^ ^]^ PDZ domains are small, globular interaction modules commonly found in scaffold proteins, and their arrangement in tandem appears to reflect a naturally evolved strategy to achieve multi‐functionality.^[^ ^20^, ^21^, ^22^, ^23^ ^]^ While PDZ domains are capable of independent folding and often found as individual domains, their frequent organization in tandem often leads to complex behaviors such as the formation of misfolded intermediates^[^ ^13^, ^16^, ^18^ ^]^ or cooperative transitions. These effects are frequently driven by transient interdomain interactions, which can stabilize non‐native states or modulate folding pathways.^[^ ^16^, ^17^, ^18^, ^19^, ^24^ ^]^ Notably, some of these misfolded states retain physiological binding activity,^[^ ^13^, ^19^ ^]^ suggesting that functional output may arise not only from the native conformation but also from alternative structural ensembles.

Despite substantial progress on our understanding of protein folding, the structural features of transient misfolded intermediates, and in particular in multidomain proteins, remains essentially unresolved. Kinetic traps have been inferred across diverse systems—from repeat proteins to immunoglobulin‐like domains, spectrin/titin modules, SH3 and PDZ tandems—using chevron curvature, burst‐phase signals, H/D exchange, relaxation dispersion, and single‐molecule experiments.^[^ ^12^, ^25^, ^26^, ^27^, ^28^, ^29^ ^]^ In these contexts, mechanistic explanations have focused on transient domain swapping, which were proposed to be at the origin of non‐native interdomain contacts and may emerge early along the pathway.^[^ ^12^, ^25^, ^30^, ^31^ ^]^ Yet, most evidence is indirect, and we still lack residue‐level energetic maps of such misfolded ensembles. Classical Φ‐value analysis has been extraordinarily powerful for native transition states in single‐domain proteins, but its application to non‐native intermediates—particularly in the presence of interdomain coupling—has been rare and methodologically challenging.^[^ ^16^, ^32^ ^]^ This gap limits our ability to connect phenomenology (biphasic kinetics, negative or anomalous Φ‐values) to a concrete structural picture.

A complementary, long‐standing issue concerns the denatured state. Residual structure and chain compaction are known to modulate nucleation, route selection, and frustration,^[^ ^33^, ^34^, ^35^, ^36^ ^]^ but comparative tests are difficult across unrelated proteins because sequence and architecture confound interpretation. It would be therefore desirable to identify tractable models allowing a controlled comparison between folding from a conformationally restrained and a free denatured ensemble within the same polypeptide, thereby disentangling the influence of denatured‐state constraints from sequence‐specific effects.

Such a system is found in the PDZ tandem repeat of the protein X11, which stands out as particularly fascinating, due to its unexpectedly high degree of folding cooperativity and the mechanistic insights it offers.^[^ ^17^, ^24^, ^37^ ^]^ Unlike other tandems where domains fold largely independently, X11 PDZ1–PDZ2 folds and unfolds as a single cooperative unit, despite each domain being individually capable of autonomous folding. This cooperativity is critically enforced by a short C‐terminal autoinhibitory segment that couples the two domains, enabling long‐range communication and structural interdependence.^[^ ^37^ ^]^ Beyond this, kinetic analyses have revealed that folding and unfolding follow distinct pathways.^[^ ^24^ ^]^ Finally, refolding experiments have uncovered the transient accumulation of a misfolded intermediate, a recurrent theme in multi‐domain folding,^[^ ^6^, ^7^, ^11^, ^12^, ^13^, ^14^, ^16^, ^18^, ^25^, ^30^, ^38^ ^]^ which highlights the potential for kinetic traps even in well‐behaved, globular systems.

To investigate the structural features of these events, we report here a detailed mutational analysis of these three interrelated features—cooperativity, pathway asymmetry, and transient misfolding. By systematically probing the energetic and kinetic effects of point mutations, we characterize the structural organization of the transition state and unfolding intermediates. Furthermore, we successfully describe the overall properties of the misfolded species encountered during refolding, revealing how subtle interdomain frustration can reshape the folding landscape of a multidomain protein.

## 2 Results and Discussion

To elucidate the molecular determinants of the folding mechanism of X11 PDZ1–PDZ2, we undertook a comprehensive Φ‐value analysis aimed at dissecting the structural features of transition states, as well as the nature of the intermediates transiently populated during folding.^[^ ^39^, ^40^, ^41^ ^]^ Our goal was to define how local interactions contribute to the overall folding cooperativity, to clarify the extent of structural formation in key metastable states, and to assess how supradomain coupling affects the folding energy landscape.

**Figure 1:**
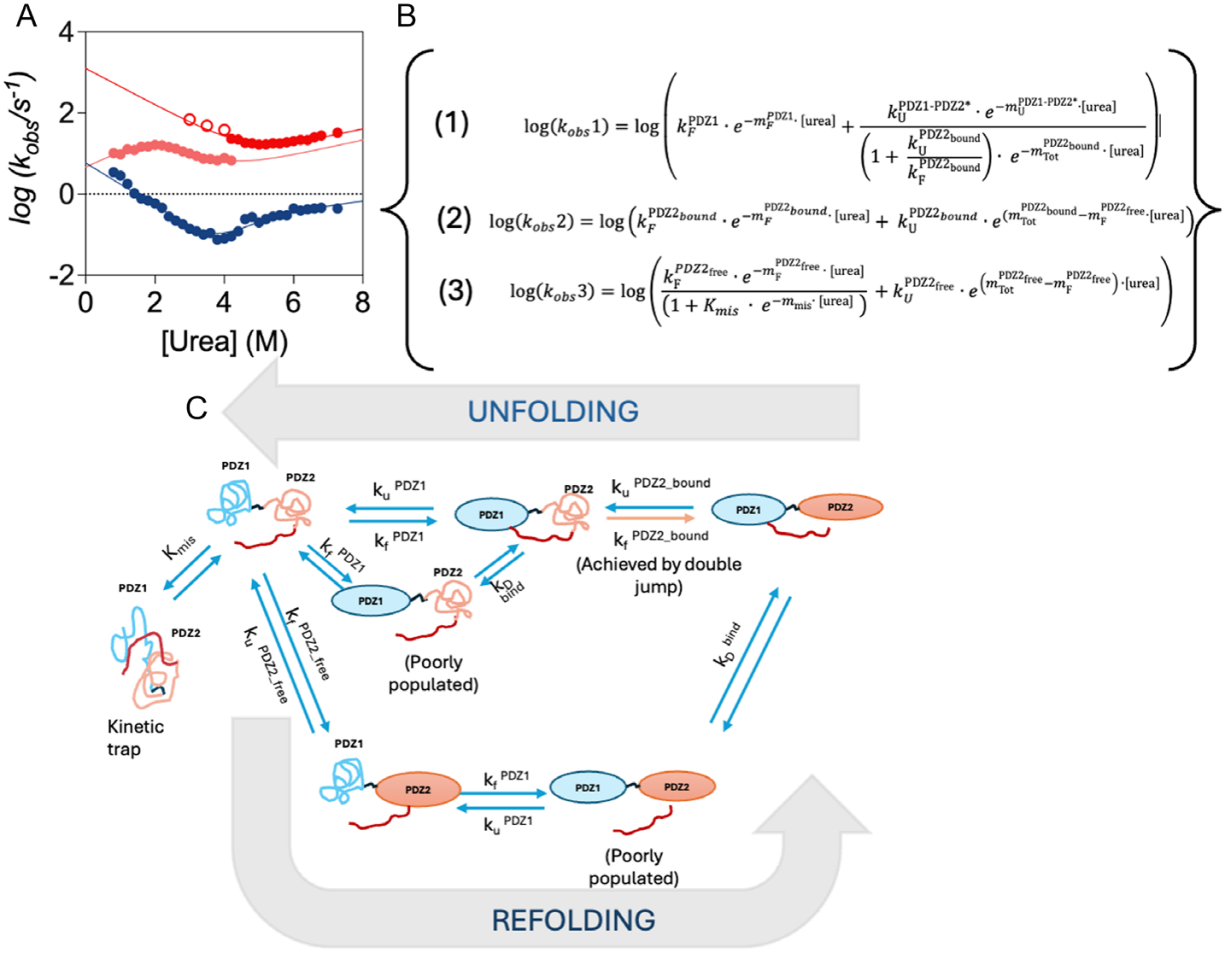
The folding and unfolding of X11 PDZ1–PDZ2. Kinetic characterization and folding scheme of the X11 PDZ1–PDZ2 tandem. Panel A shows the chevron plot for X11 PDZ1–PDZ2 obtained from kinetic experiments using single‐jump (filled symbols) and double‐jump (open symbols) protocols, as described in ref. [17, 24]. In a chevron plot, the logarithm of the observed rate constants is plotted as a function of denaturant concentration. The left‐ and right‐hand limbs correspond respectively to folding and unfolding transitions, and deviations from linearity reveal the accumulation of kinetic intermediates. The plot displays a biphasic behavior at all urea concentrations and a pronounced downward curvature in the folding limb of PDZ2 (red circles), indicative of the accumulation of a misfolded intermediate. The lines are the best fit to the model depicted in Panel B (blue, equation 1; red, equation 2; orange, equation 3). Panel B reports the kinetic model used to interpret the data, consisting of parallel folding and unfolding of the PDZ domains, as described in detail in ref. [17, 24]. Rate constants for interconversion between native (N), intermediate (I), and denatured (D) states are defined as in the original model. Panel C presents a schematic structural representation of the folding and unfolding reactions, adapted from the same reference, illustrating the early folding of PDZ2, delayed folding of PDZ1, and the regulatory role of the C‐terminal tail. The scheme captures the structural asymmetry and interdomain coordination that dictates the folding landscape of the tandem.

Because of the exceptional complexity of the folding and unfolding pathways of this system, it is appropriate to first recapitulate the main features and quantitative aspects of the underlying kinetic scheme and, only then, introducing the mutational data. A critical element in X11 PDZ1–PDZ2 folding is represented by the short C‐terminal tail, which acts as an intramolecular ligand of PDZ1^[^ ^37^ ^]^ and plays a central role in mediating the cooperative behavior observed during unfolding.^[^ ^17^, ^24^ ^]^ In this direction, unfolding proceeds via an ordered pathway in which PDZ2 denatures first, while PDZ1 remains folded and continues to bind the tail, delaying its release until late in the process. This results in a relatively well‐coupled mechanism, stabilized by interdomain interactions. In refolding, however, the situation is strikingly different: PDZ2 refolds more rapidly and largely independently of PDZ1, while productive tail binding can only occur once PDZ1 has also folded.^[^ ^24^ ^]^ This leads to a looser coupling between the two domains during folding and allows the transient accumulation of a misfolded intermediate that competes with the native state. This mechanistic asymmetry is reflected in the biphasic behavior observed in both folding and unfolding kinetics, indicating that distinct structural species populate the reaction coordinate in each direction. A summary of the minimal kinetic folding mechanism of X11 PDZ1–PDZ2 together with its associated equations is reported in **Figure** **1**.

### 2.1 Deciphering the Structural Features of the Metastable States in the Folding of X11 PDZ1‐PDZ2

To probe the structural features of such intermediates and transition states, we applied Φ‐value analysis, a well‐established method combining site‐directed mutagenesis with kinetic measurements.^[^ ^39^, ^40^, ^41^ ^]^ This approach enables the identification of native‐like structural elements at specific positions by comparing the free energy changes induced by a mutation in a metastable state to those in the native state. In short, the Φ‐value represents a normalized measure of native‐like structure at the site of mutation: a value of 1 indicates full structuring in the metastable state, a value of 0 indicates full disorder, and intermediate values reflect partial structuring. We designed 48 single‐site variants of X11 PDZ1–PDZ2 following standard criteria for Φ‐value analysis, introducing conservative truncations (typically Leu → Ala, Ile → Val, Val → Ala, Thr → Ser, Ala → Gly substitutions) at hydrophobic core residues, which are least likely to perturb the overall structure and folding mechanism.^[^ ^39^, ^41^ ^]^ All variants were subjected to a full kinetic characterization through stopped‐flow experiments, including both single‐jump (folding and unfolding) and double‐jump (refolding) protocols, allowing a detailed and quantitative description of the folding landscape of this multidomain system. The detailed description of the experimental set‐up is reported in the Materials and Methods section.

**Figure 2:**
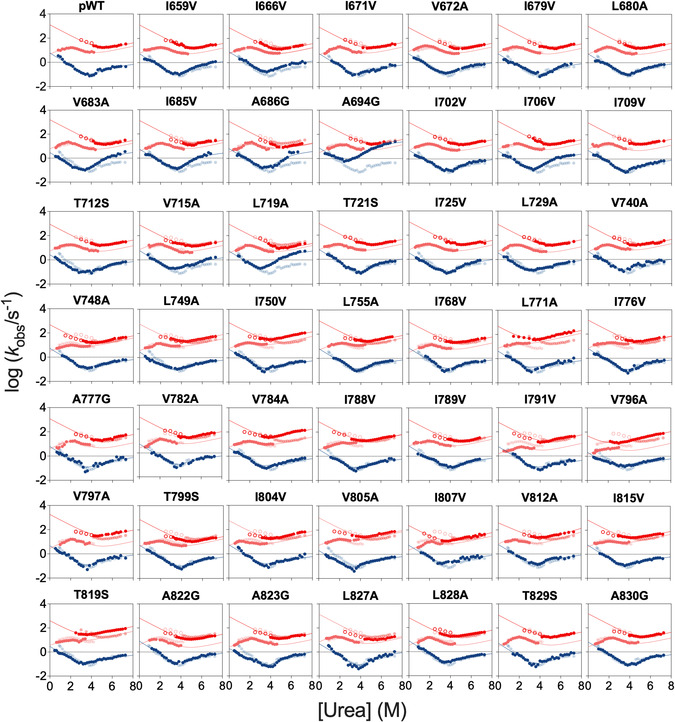
Kinetic chevron plots for wild‐type and mutant variants of X11 PDZ1–PDZ2. In analogy to what indicated in Figure 1, data obtained from double jump are reported as open circles. In all panels, wild‐type data are overlaid in a semi‐transparent style to serve as a reference for comparison with each mutant. The lines are the best fit to the model depicted in Panel 1B (blue, equation 1; red, equation 2; orange, equation 3).

**Figure 3:**
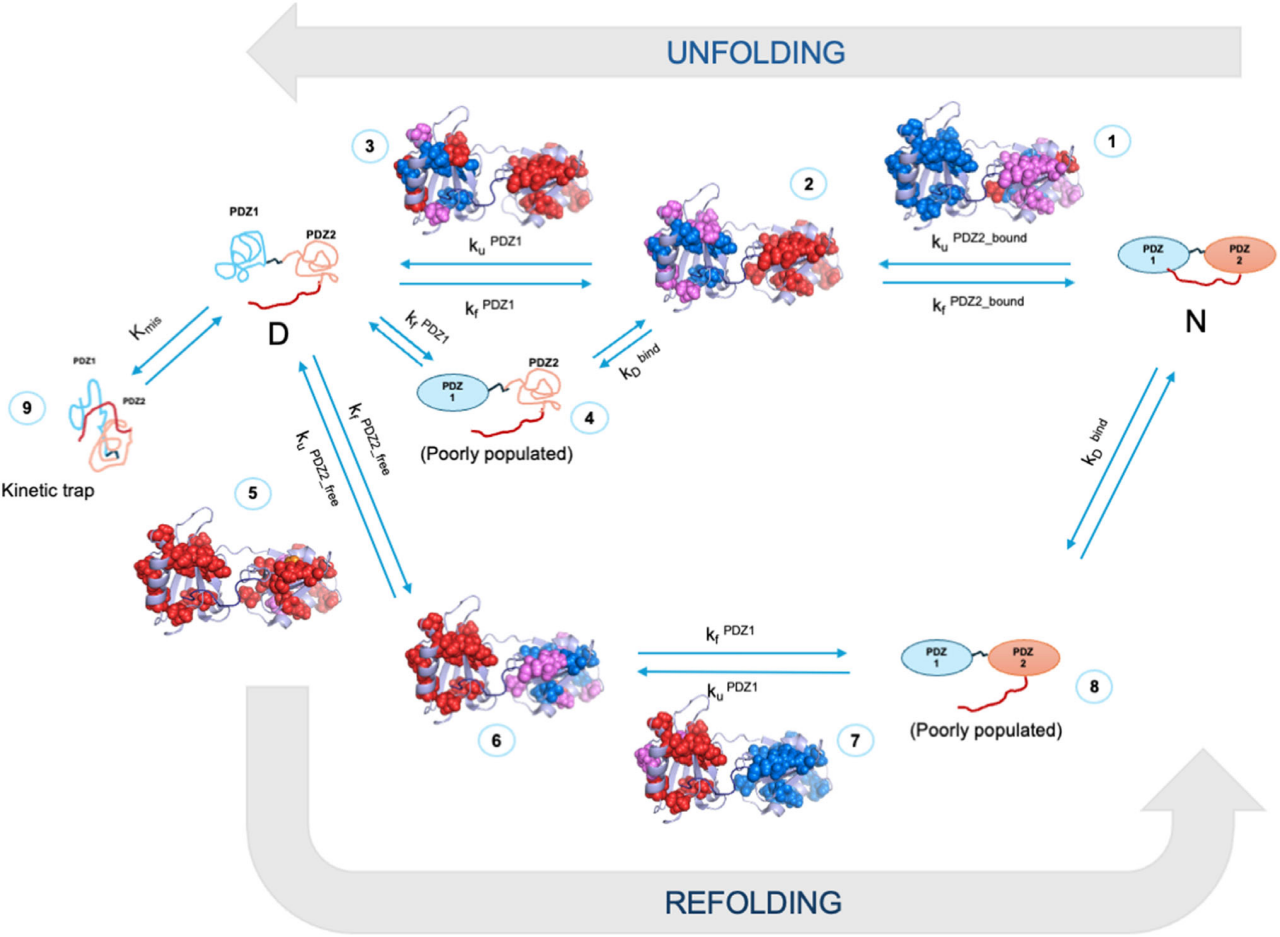
The folding and unfolding pathways of X11 PDZ1–PDZ2 as obtained from Φ‐value analysis. The measured Φ values of each of the experimentally addressable state are mapped on to the structure of the tandem. Residues are color‐coded: Φ < 0.3 (red), 0.3–0.7 (magenta), Φ > 0.7 (blue). In analogy to standard rules of Φ‐value analysis,^[^ ^39^, ^40^, ^41^ ^]^ the mutants were divided in three groups based on their measured Φ values: small (Φ < 0.3; red), intermediate (0.3 < Φ < 0.7; magenta), and large (Φ > 0.7; blue).

**Figure** **2** summarizes all the kinetic experiments carried out on the different variants compared to that of the wild‐type protein. As expected, in all cases, both folding and unfolding experiments were consistent with a double exponential decay, which could be readily characterized at different denaturant concentrations. Data were then fitted to the model depicted in Figure 1 and the calculated folding and unfolding parameters are reported in **Table** **1** and S1, Supporting Information. By following the standard rules of Φ‐value analysis,^[^ ^39^, ^40^, ^41^ ^]^ we then normalized the changes in free energy upon mutagenesis of each of the experimentally accessible state to that of the native state. To provide structural information of the intermediate and transition states, the mutants were divided in three groups based on their measured Φ values: small (Φ < 0.3; red), intermediate (0.3 < Φ < 0.7; magenta), and large (Φ > 0.7; blue). The color‐coded mutations were then mapped onto the structure of X11 PDZ1–PDZ2 (**Figure** **3**). A more detailed depiction of the different states is also reported in Figure S1, Supporting Information.

**Table 1:** Thermodynamic parameters of PDZ1‐PDZ2 variants.

	ΔΔ*G*_D‐N_ [kcal mol^−1^]	Φ_D‐1_	Φ_D‐2_	Φ_D‐3_	Φ_D‐5_	Φ_D‐6_	Φ_D‐7_	Φ_mis_
pWT	–	–	–	–	–	–	–	–
I659V	0.48 ± 0.05	1.07 ± 0.08	0.95 ± 0.10	0.70 ± 0.10	0.010 ± 0.02	−0.07 ± 0.02	0.25 ± 0.02	0.23 ± 0.1
I666V	0.57 ± 0.05	1.07 ± 0.08	0.42 ± 0.05	0.70 ± 0.04	−0.010 ± 0.02	−0.08 ± 0.02	−0.28 ± 0.01	0.11 ± 0.1
I671V	0.40 ± 0.06	0.84 ± 0.04	−0.10 ± 0.01	−0.18 ± 0.01	−0.020 ± 0.04	0.14 ± 0.05	0.17 ± 0.01	−0.45 ± 0.04
V672A	0.55 ± 0.07	1.05 ± 0.10	0.40 ± 0.02	0.30 ± 0.01	−0.030 ± 0.02	−0.08 ± 0.05	0.10 ± 0.01	0.37 ± 0.09
I679V	0.07 ± 0.04	–	–	–	–	–	–	–
L680A	0.33 ± 0.05	–	–	–	–	–	–	–
V683A	1.04 ± 0.15	1.18 ± 0.10	1.26 ± 0.15	0.84 ± 0.10	0.030 ± 0.002	−0.15 ± 0.04	0.41 ± 0.02	0.070 ± 0.1
I685V	0.70 ± 0.08	1.22 ± 0.10	1.28 ± 0.10	1.20 ± 0.20	−0.010 ± 0.002	−0.22 ± 0.05	0.07 ± 0.02	−0.01 ± 0.08
A686G	0.51 ± 0.04	1.08 ± 0.10	1.15 ± 0.10	1.05 ± 0.05	0.15 ± 0.01	−0.20 ± 0.02	0.11 ± 0.01	−0.09 ± 0.09
A694G	2.06 ± 0.10	1.01 ± 0.10	0.97 ± 0.06	1.05 ± 0.20	−0.010 ± 0.003	−0.020 ± 0.02	−0.09 ± 0.00	−0.16 ± 0.09
I702V	0.38 ± 0.03	–	–	–	–	–	–	–
I706V	0.91 ± 0.02	1.05 ± 0.10	0.98 ± 0.05	0.83 ± 0.04	0.12 ± 0.02	0.070 ± 0.05	0.15 ± 0.01	0.24 ± 0.09
I709V	0.38± 0.05	–	–	–	–	–	–	–
T712S	0.83 ± 0.03	0.94 ± 0.05	0.61 ± 0.01	0.02 ± 0.01	−0.05 ± 0.02	0.010 ± 0.03	0.58 ± 0.02	0.01 ± 0.09
V715A	0.80 ± 0.02	1.07 ± 0.04	0.60 ± 0.03	0.57 ± 0.01	0.17 ± 0.03	0.100 ± 0.02	0.030 ± 0.01	0.30 ± 0.02
L719A	1.25 ± 0.10	1.27 ± 0.10	1.05 ± 0.10	1.0 ± 0.30	0.02 ± 0.02	−0.250 ± 0.02	0.040 ± 0.02	−0.14 ± 0.04
T721S	−0.26 ± 0.08	–	–	–	–	–	–	–
I725V	0.53 ± 0.06	0.9 ± 0.06	0.99 ± 0.10	0.34 ± 0.02	0.16 ± 0.01	0.050 ± 0.02	0.64 ± 0.02	0.43 ± 0.02
L729A	0.8 ± 0.08	1.05 ± 0.10	0.52 ± 0.02	0.15 ± 0.02	0.11 ± 0.03	0.16 ± 0.02	0.37 ± 0.05	0.09 ± 0.09
V740A	0.92 ± 0.08	1.07 ± 0.15	0.42 ± 0.02	0.25 ± 0.03	−0.11 ± 0.02	−0.040 ± 0.02	0.17 ± 0.02	0.01 ± 0.02
V748A	1.04 ± 0.10	0.76 ± 0.05	−0.02 ± 0.001	−0.08 ± 0.02	0.52 ± 0.04	0.76 ± 0.10	0.94 ± 0.07	0.01 ± 0.04
L749A	1.42 ± 0.20	0.69 ± 0.02	0.26 ± 0.02	−0.06 ± 0.01	0.16 ± 0.01	0.47 ± 0.05	0.68 ± 0.05	−0.07 ± 0.09
I750V	1.26 ± 0.10	0.37 ± 0.02	−0.06 ± 0.01	−0.13 ± 0.02	0.13 ± 0.01	0.76 ± 0.02	0.94 ± 0.10	0.07 ± 0.09
L755A	0.66 ± 0.09	0.35 ± 0.04	−0.02 ± 0.01	−0.01 ± 0.02	−0.010 ± 0.02	0.64 ± 0.02	1.00 ± 0.10	−0.26 ± 0.04
V763A	1.90 ± 0.20	0.55 ± 0.01	0.29 ± 0.02	−0.05 ± 0.00	0.37 ± 0.04	0.82 ± 0.01	0.66 ± 0.02	0.11 ± 0.05
I768V	0.50 ± 0.09	0.52 ± 0.02	−0.10 ± 0.01	−0.020 ± 0.002	0.26 ± 0.02	0.74 ± 0.05	1.05 ± 0.10	−0.50 ± 0.06
L771A	1.90 ± 0.15	0.45 ± 0.01	0.09 ± 0.01	−0.01 ± 0.01	0.24 ± 0.02	0.79 ± 0.03	0.89 ± 0.05	0.14 ± 0.04
I776V	0.40 ± 0.03	0.23 ± 0.01	−0.05 ± 0.02	0.02 ± 0.02	0.13 ± 0.02	0.90 ± 0.01	1.08 ± 0.10	0.22 ± 0.05
A777G	0.45 ± 0.02	0.43 ± 0.01	0.03 ± 0.01	0.05 ± 0.002	−0.74 ± 0.04	−0.17 ± 0.04	1.05 ± 0.10	‐0.15 ± 0.05
V782A	0.52 ± 0.07	0.46 ± 0.02	0.25 ± 0.02	−0.30 ± 0.02	−0.62 ± 0.05	−0.09 ± 0.02	0.95 ± 0.05	−1.47 ± 0.10
V784A	1.62 ± 0.20	0.42 ± 0.03	0.01 ± 0.01	−0.07 ± 0.02	0.080 ± 0.03	0.66 ± 0.02	0.96 ± 0.03	0.05 ± 0.02
I788V	1.14 ± 0.10	0.74 ± 0.03	0.08 ± 0.02	−0.16 ± 0.03	0.21 ± 0.01	0.47 ± 0.01	0.75 ± 0.05	−0.48 ± 0.03
I789V	0.30 ± 0.06	–	–	–	–	–	–	–
I791V	0.92 ± 0.09	0.72 ± 0.05	0.36 ± 0.03	−0.39 ± 0.03	0.74 ± 0.04	1.01 ± 0.10	0.97 ± 0.02	−0.17 ± 0.02
V796A	1.96 ± 0.20	0.68 ± 0.02	0.37 ± 0.01	−0.69 ± 0.05	0.39 ± 0.03	0.70 ± 0.02	1.00 ± 0.10	−0.1 ± 0.1
V797A	0.77 ± 0.06	0.33 ± 0.01	0.21 ± 0.01	0.03 ± 0.03	−0.15 ± 0.01	0.52 ± 0.02	0.81 ± 0.10	−0.62 ± 0.08
T799S	0.24 ± 0.01	–	–	–	–	–	–	–
I804V	1.05 ± 0.07	0.62 ± 0.02	−0.08 ± 0.02	−0.12 ± 0.03	0.73 ± 0.03	1.11 ± 0.10	0.96 ± 0.02	0.65 ± 0.04
V805A	1.57 ± 0.10	0.65 ± 0.02	0.35 ± 0.02	−0.07 ± 0.02	0.11 ± 0.01	0.46 ± 0.05	0.57 ± 0.04	−0.03 ± 0.4
I807V	1.51 ± 0.10	0.83 ± 0.05	0.08 ± 0.02	0.05 ± 0.04	−0.15 ± 0.01	0.01 ± 0.02	0.97 ± 0.05	−0.50 ± 0.05
V812A	1.13 ± 0.2	0.54 ± 0.02	0.01 ± 0.02	0.01 ± 0.01	0.31 ± 0.03	0.77 ± 0.02	1.00 ± 0.10	0.24 ± 0.06
I815V	0.95 ± 0.07	0.53 ± 0.03	−0.13 ± 0.01	−0.14 ± 0.02	0.44 ± 0.02	0.91 ± 0.05	0.99 ± 0.05	0.10 ± 0.06
T819S	1.77 ± 0.10	0.60 ± 0.05	0.15 ± 0.02	−0.09 ± 0.02	0.33 ± 0.02	0.75 ± 0.05	0.76 ± 0.10	0.05 ± 0.05
A822G	0.20 ± 0.06	–	–	–	–	–	–	–
A823G	0.29 ± 0.08	–	–	–	–	–	–	–
L827A	−0.10 ± 0.08	–	–	–	–	–	–	–
L828A	0.60 ± 0.05	0.57 ± 0.03	0.33 ± 0.02	−0.04 ± 0.02	0.22 ± 0.04	0.65 ± 0.05	1.3 ± 0.1	−0.21 ± 0.02
T829S	0.43 ± 0.08	–	–	–	–	–	–	–
A830G	−0.10 ± 0.09	–	–	–	–	–	–	–

Kinetic parameters were obtained by globally fitting the data in Figure 2 to the system of equations reported in Figure 2B with shared *m*‐values. Errors reported are standard errors. The equations used are reported in the Materials and Methods section. By following standard rules of Φ values, mutations leading a change in stability of less than 0.4 kcal mol^−1^ were excluded from the analysis.

An overall inspection of Figure 3 confirms what previously proposed for the folding mechanism of X11 PDZ1‐PDZ2 and summarized schematically in Figure 1. In fact, in the unfolding pathway, it is observed that mutations in PDZ1 tend to display high values of Φ in the intermediate (denoted as state **2** in Figure 3), indicating native‐like structure, whereas PDZ2 shows low Φ values, corresponding to a denatured‐like ensemble. Conversely, the refolding mechanism is markedly different due to the lack of interaction between the C‐terminal autoinhibitory tail and PDZ1, which cannot occur until the latter is folded. In fact, in the case of refolding, the reaction proceeds with the early structure formation of PDZ2 (state **6** in Figure 3), followed by the formation of PDZ1 with concurrent binding of the autoinhibitory tail. Hence, for the sake of clarity we will discuss the structural features of the two pathways separately.

### 2.2 The Unfolding of X11 PDZ1‐PDZ2

The unfolding trajectory of X11 PDZ1–PDZ2 (top line in Figure 3), as revealed by Φ‐value analysis, highlights a highly asymmetric denaturation mechanism dominated by the persistent structuring of PDZ1 and the early destabilization of PDZ2. Specifically, mutations insisting on the PDZ1 domain consistently yielded high Φ values (depicted in magenta and in blue in Figure 3 and Figure S1, Supporting Information). In the case of PDZ2, we observed higher Φ values in residues located near the interface between strands β2 and β3, with further support from residues located in α2; high values were also detectable in the region between β1 and β6 suggesting that also these elements remain structurally intact during the initial stages of unfolding. As expected, PDZ1 retains a native‐like fold in the intermediate state populated en route to complete denaturation (state **2**), and the folding nucleus between strands β2, β3 and α2 (with support from interactions with α1) may also be observed as structured at the late stages of unfolding (state **3**).

In contrast, PDZ2 exhibits markedly lower Φ values across the whole unfolding pathway, indicating that it denatures early and contributes little native‐like structure to the unfolding intermediate. This differential behavior between the two domains supports the previous suggestion that unfolding proceeds in an ordered fashion—PDZ2 unfolds first, while PDZ1 persists in a quasinative conformation stabilized by its interaction with the C‐terminal tail.^[^ ^24^ ^]^ The autoinhibitory tail continues to engage PDZ1 until late in the denaturation process, effectively delaying its full unfolding. This mechanism reinforces the view that cooperative interactions between the two domains—particularly the intramolecular binding of the tail to PDZ1—confer a degree of kinetic control that shapes the reaction pathway.^[^ ^9^, ^31^ ^]^

Notably, residues at the domain interface and within the tail‐binding pocket of PDZ1 displayed the highest Φ values (see states **2** and **3** in Figure 3 and S1, Supporting Information), underscoring their pivotal role in maintaining structural integrity during unfolding. Meanwhile, residues in more peripheral regions of PDZ1 showed intermediate or fractional Φ values, suggesting a gradual loss of structure that initiates only after PDZ2 has denatured.

Taken together, these observations establish a model in which unfolding is not a symmetric reversal of folding, but instead a sequential process driven by interdomain coupling and modulated by the specific structural persistence of PDZ1. This mechanistic asymmetry further might reflect the functional specialization of the tandem, where the activity of PDZ1 is linked to the internal ligand release, physically linked to PDZ2.^[^ ^8^, ^37^ ^]^

### 2.3 The Folding of X11 PDZ1‐PDZ2

The folding pathway of X11 PDZ1–PDZ2 (bottom line in Figure 3) presents a markedly different mechanism from its unfolding, defined by the parallel yet kinetically distinct folding of the two constituent domains. In fact, PDZ2 folds rapidly and largely independently from PDZ1. The values of Φ suggest early weak native‐like structure formation at the interface between β1, β6 and α2 in the transition state of folding (state **5** in Figure 3 and S1, Supporting Information), which is consequently consolidated throughout the whole globule (state **6** in Figure 3 and S1, Supporting Information). PDZ1, in contrast, folds more slowly, as reflected by a broader range of Φ values and the absence of high native‐like structure in early intermediates. Φ values appear generally very low and broadly distributed within the structure (state **7** in Figure 3 and S1, Supporting Information). Both PDZ1 and PDZ2 appear consistent with an overall behavior that recalls the so‐called nucleation‐condensation mechanism, characterized by the presence of a weak nucleus and concomitant global collapse.^[^ ^42^, ^43^ ^]^ These data imply that PDZ1 formation lags behind PDZ2, and that the formation of its folding nucleus occurs later in the folding process.

Importantly, the short C‐terminal autoinhibitory tail—a critical element for domain coupling in the native state—can only engage PDZ1 once the domain is fully or near‐fully folded. As such, tail binding is restricted to a downhill event following the main folding barrier of PDZ1 (state **8** in Figure 3 and S1, Supporting Information). This condition results in a looser temporal coupling between the domains during folding compared to unfolding, with the domains folding through partially independent parallel pathways. Only upon folding of PDZ1 can the tail binding contribute to stabilizing the final native state, enforcing the cooperative features observed at equilibrium.^[^ ^17^ ^]^

This mechanistic arrangement explains the observed biphasic folding and unfolding kinetics and the transient population of misfolded intermediates. The interplay between fast PDZ2 folding, slow PDZ1 folding, and delayed tail binding thus expands the conformational landscape and introduces complexity in the folding trajectory that are not present in the unfolding process.

Taken together, these findings reveal that folding proceeds via parallel pathways wherein the two domains fold independently but converge structurally upon completion of PDZ1 folding and subsequent tail binding. This asymmetry not only contrasts the ordered, sequential nature of unfolding but also highlights the role of intramolecular interactions in modulating pathway choice and domain communication during protein self‐assembly.

### 2.4 Probing the Role of Denatured State Structure in PDZ2 Folding

Several studies have emphasized the importance of residual structure of denatured states in shaping the folding pathways of proteins.^[^ ^33^, ^34^, ^36^, ^44^, ^45^, ^46^, ^47^, ^48^, ^49^ ^]^ However, a direct comparison of folding from distinct denatured ensembles within the same protein system and under similar experimental conditions is particularly challenging. In this context, the PDZ2 domain of X11 provides a unique opportunity. In fact, the Φ‐value analysis of its folding kinetics allows for the comparison of two distinct denatured‐state scenarios within the same construct and dataset.

In this context, it is important to clarify the structural definition of the “restricted denatured state” referred to throughout this study. This ensemble corresponds specifically to the population observed during kinetic unfolding, when PDZ1 remains folded and bound to the C‐terminal tail while PDZ2 is denatured. This interpretation is directly supported by the outcome of the double‐jump experiments, in which the monitored refolding phase reports exclusively on PDZ2, with no detectable contribution from PDZ1, thus unequivocally indicating that PDZ1 remains folded under these conditions. Under strongly denaturing conditions, this residual interaction is fully disrupted, and both domains unfold cooperatively, as previously demonstrated.^[^ ^17^, ^24^ ^]^ The restricted denatured state therefore represents a transient, partially unfolded ensemble, not an incomplete global denaturation, and its experimental delineation provides the mechanistic basis for dissecting the individual kinetic steps resolved in this work. More to the point, during standard refolding experiments, PDZ2 initiates folding from a disordered and unconstrained chain, resembling a canonical denatured ensemble. In contrast, in the unfolding intermediate (state **2** in Figure 3), the denatured state is conformationally restrained due to the intramolecular binding of the C‐terminal autoinhibitory tail with the folded PDZ1. This intramolecular interaction limits the conformational entropy of the PDZ2‐unfolded ensemble, possibly introducing specific contacts or transient structure.

**Figure 4:**
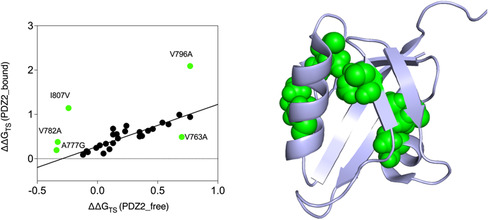
Comparison of the folding transition state of folding between free and restrained (C‐terminal bound) denatured state of PDZ2. It is evident that, while the majority of the mutants display a conserved ΔΔG_D‐TS_ when the two conditions are compared, some relevant outliers may be identified. These outliers are indicated in green in the graph and mapped on to the structure of PDZ2.

To quantitatively assess how these differences in denatured‐state structure affect the folding pathway of PDZ2, we compared the mutation‐induced changes in activation free energy (ΔΔ*G*_D‐TS_) derived from refolding (unconstrained denatured state) and unfolding/double jump experiments (conformationally restrained denatured state) experiments, i.e., the folding behavior of PDZ2 in states **1** and **5** in Figure 3. This comparison is illustrated in **Figure** **4**A.

The resulting correlation reveals a predominantly linear relationship, indicating that for most of the probed positions, the energetic contribution of the mutated residue to the transition state is largely insensitive to the state of the denatured ensemble. However, a distinct cluster of residues deviates significantly from this trend, represented by residues V763, A777, V782, V796, and I807. These outliers are characterized by a different ΔΔ*G*_D‐TS_ values in the free and constrained denatured states, suggesting that these residues participate in transient interactions or local structure formation in the restrained denatured state that are absent under refolding conditions.

These findings support the hypothesis that structural bias in the unfolded ensemble can finely modulate the apparent folding mechanism, even in a domain that is otherwise capable of autonomous folding. Moreover, they identify specific regions of PDZ2, located at the interface between α1, β2, α2, likely involved in early non‐native or frustrated contacts when the denatured state is conformationally restricted.

More broadly, these experiments provide a rare opportunity to compare folding from a conformationally restrained versus a free denatured ensemble within the same polypeptide chain. Such a direct comparison is seldom possible across unrelated proteins, where sequence and architectural differences confound interpretation. The X11 tandem thus offers a unique model to evaluate how denatured‐state constraints influence nucleation and pathway selection, reinforcing the view that residual structure in the denatured ensemble can directly modulate folding energetics and pathway outcomes.

### 2.5 Comparing the Folding Mechanism of Isolated Domains to that of the Tandem

An important question in the study of multidomain protein folding is whether the folding mechanisms of individual domains remain conserved when examined in isolation or whether they are altered in the context of more complex, multidomain assemblies. Hence, we expressed and purified PDZ1 and PDZ2 in isolation and subjected them to Φ‐value analysis. Twenty mutants on PDZ1 and Twenty‐nine mutants for PDZ2 were obtained and fully characterized. Figure S2 and Table S2, Supporting Information, summarize the kinetic analysis of all the variants compared to their respective isolated PDZ domains.

**Figure 5:**
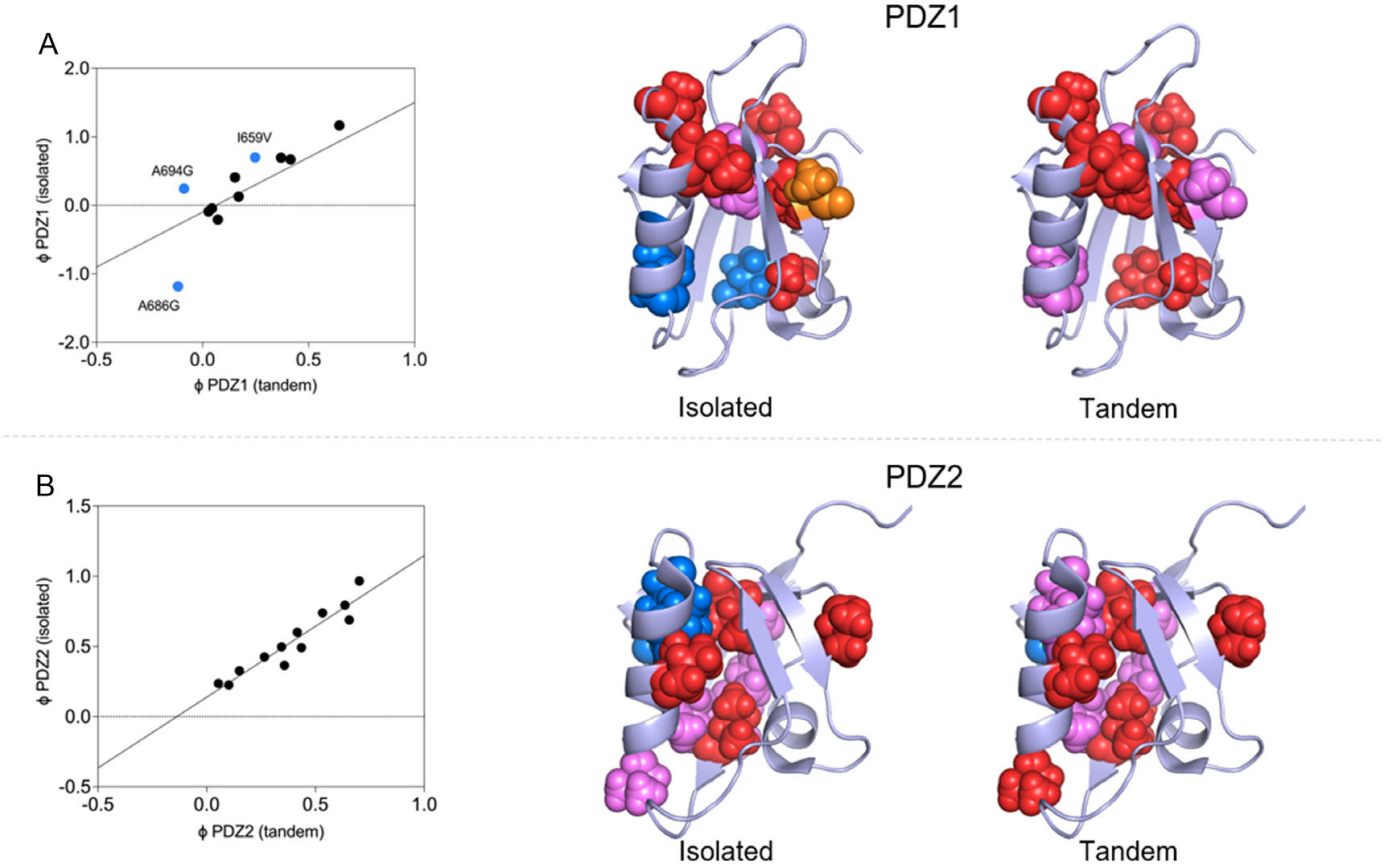
Comparison of the folding pathway of protein domains in isolation and in the context of the tandem. Panels A and B refer to PDZ1 and PDZ2 respectively. The mutants were divided in three groups based on their measured Φ values: small (Φ < 0.3; red), intermediate (0.3 < Φ < 0.7; magenta), and large (Φ > 0.7; blue). Negative values of Φ are indicated in orange. Color‐coded mutations were then mapped onto the structure.

To assess how the folding behavior of the isolated domains compares to that of the X11 PDZ1–PDZ2 tandem, we analyzed the Φ–Φ plots of homologous variants derived from the different constructs (**Figure** **5**A,B). In parallel, Φ‐values were mapped onto the corresponding structures using the same color‐coding criteria described previously. Inspection of Figure 5 clearly reveals a robust consistency in the Φ‐values obtained for both PDZ1 and PDZ2 across constructs, with strong linear correlations observed between the isolated domains and their tandem counterparts. This stark agreement suggests that, despite differences in structural context, the overall folding mechanisms of the two domains are preserved and intrinsically robust.

We note that the correlation is particularly robust in the case of PDZ2, where the Φ‐values show minimal deviation. In contrast, a subset of residues in PDZ1 appear as reproducible outliers. Notably, many of these deviations are located near the ligand‐binding pocket of the domain, which in the molecular orientation adopted in Figure 5 is positioned front‐facing, toward the viewer, between helix α2 and strand β3. This observation is particularly meaningful given the functional role of the C‐terminal autoinhibitory tail, which binds to PDZ1 in the tandem construct but is absent in the isolated domain. In the context of the tandem, Φ‐values reflect folding behavior under autoinhibited conditions, where tail binding may prestructure the binding groove or impose allosteric constraints. In contrast, the isolated domain folds in the absence of this regulatory interaction, leading to differences in the local folding energetics at or near the functional interface. Thus, the apparent disagreement in Φ‐values is not indicative of experimental noise, but rather reflects real, context‐dependent differences in folding that arise from functional regulation.

Previous theoretical and experimental works have suggested that functional regions within proteins often coincide with areas of local energetic frustration, with competing energetic needs between folding and function.^[^ ^50^, ^51^, ^52^, ^53^, ^54^, ^55^, ^56^ ^]^ These frustrated regions are typically more malleable, supporting conformational flexibility that is essential for activity. From a folding perspective, these regions may thus deviate from the global folding pathway, exhibiting delayed structuring or variable Φ‐values depending on context.^[^ ^16^, ^52^, ^53^, ^57^ ^]^ By following this view, the minor but detectable context‐dependent folding behavior observed in PDZ1 in functional regions likely arises from the fine balance between folding efficiency and functional adaptability in multidomain proteins and reinforce the importance of folding studies in revealing latent regulatory features within protein architecture.

### 2.6 The Structural Properties of Transient Misfolding as Probed by Φ Value Analysis

Inspection of the chevron plots for X11 PDZ1–PDZ2 reported in Figure 2 reveals a clear downward curvature in the folding limb of PDZ2, which specifically arises in the PDZ2 domain when it is studied within the tandem repeat (this feature is in fact absent in the isolated domain constructs, as illustrated in Figure S2, Supporting Information). The emergence of this curvature, previously reported in other multidomain proteins,^[^ ^6^, ^7^, ^11^, ^12^, ^13^, ^14^, ^16^, ^18^, ^25^, ^30^, ^38^ ^]^ has been ascribed to the presence of transient misfolding events and seems to represent a general property of multidomain proteins. However, to date such phenomenon has not been explored through systematic site‐directed mutagenesis.

**Figure 6:**
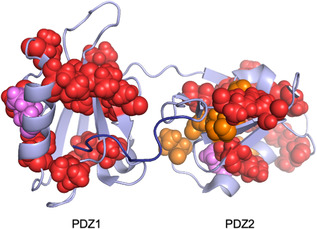
Φ‐value distribution in the misfolded intermediate of X11 PDZ1–PDZ2. The mutants were divided in three groups based on their measured Φ values: small (Φ < 0.3; red), intermediate (0.3 < Φ < 0.7; magenta), and large (Φ > 0.7; blue). Negative values of Φ are indicated in orange. Color‐coded mutations were then mapped onto the structure.

Own to the large mutational data set produced in this work, it is of special interest to discuss the structural properties of the misfolded intermediate on the light of the Φ value analysis. **Figure** **6** reports the distribution of the calculated Φ value mapped onto the structure of X11 PDZ1‐PDZ2 (data reported in Table 1). We observe that, while the misfolded intermediate appears rather insensitive to mutagenesis in PDZ1, a relevant fraction of mutants insisting on PDZ2 correspond to a detectable change in stability of kinetic trap. Of particular interest, a subset of PDZ2 mutations consisting of 5 mutants (indicated in orange in Figure 6) exhibit negative Φ‐values, which are classically interpreted as signatures of noncanonical energetic responses.^[^ ^39^, ^40^, ^41^ ^]^ These outlier residues cluster near the interface between the two domains. This spatial distribution strongly suggests that these residues play a pivotal role in stabilizing non‐native interactions within the misfolded ensemble. It is worth noting that equilibrium unfolding measurements are not informative in this system, as X11 PDZ1–PDZ2 unfolds as a single cooperative unit. Under these conditions, equilibrium ΔG values would only report on the overall transition, with both domains either simultaneously native or denatured, and cannot resolve the stepwise energetic contributions of individual domains or intermediates. By contrast, the kinetic framework used here allows estimation of all microscopic rate constants in the folding scheme, enabling Φ‐values to be calculated separately for the different steps. This stepwise resolution provides access to structural features of both productive and nonproductive intermediates that would remain invisible in equilibrium experiments.

A powerful strategy to investigate the structural features of such transiently populated states is offered by Leffler (or Bronsted) analysis, which correlates the energetic effects of perturbations, such as those introduced by point mutations, on two different conformational states.^[^ ^29^, ^58^, ^59^ ^]^ If the perturbed residues contribute similarly to both states, the resulting Bronsted plot is expected to be linear, with the slope (commonly denoted as α value) reflecting the degree of structural similarity between the two states.

**Figure 7:**
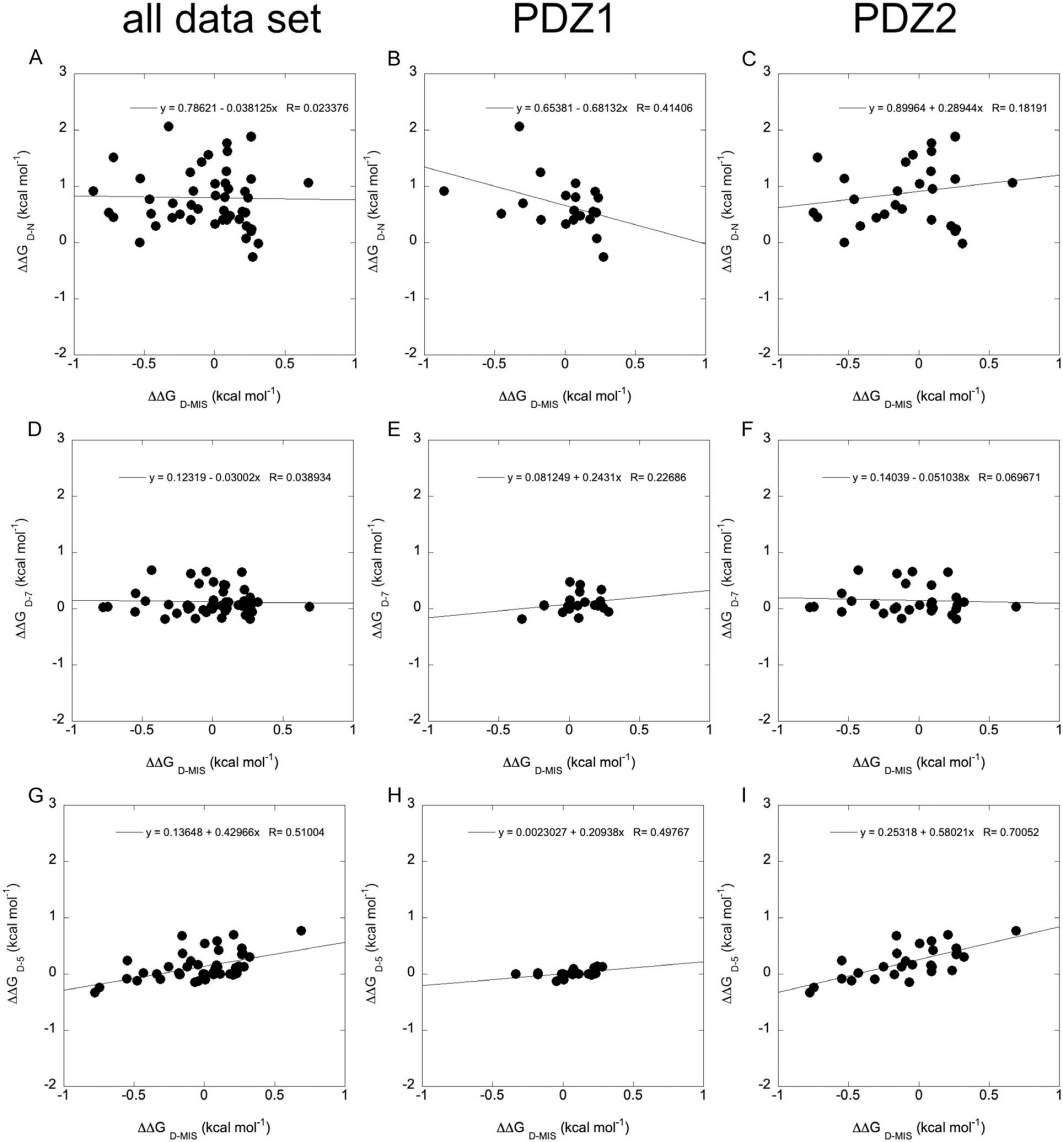
Bronsted plot analysis of the misfolded intermediate versus the native (panels A, B and C), state 7 (panels D, E, and F) and 5 (panels G, H, and I). In all cases, lines are the best fit to a linear function. The interpretation of the observed Bronsted analysis is discussed in the text.

To probe the relationship between the misfolded trap and the different states for the folding of X11 PDZ1‐PDZ2, we performed a Bronsted analysis comparing the effects of mutations on the folding barrier and the population of the misfolded intermediate. The resulting plots are shown in **Figure** **7**.

We focused this analysis on comparing the energetics of the misfolded trap with those of the native state and of the transition states involved in the folding of PDZ1 and PDZ2. Specifically, we plotted the mutation‐induced changes in free energy of the misfolded ensemble (ΔΔ*G*_D‐MIS_) against those affecting the native state, the PDZ1 folding transition state (state **7** in Figure 3), and the PDZ2 folding transition state (state **5**). The resulting correlations are shown in Figure 6. We further parsed the dataset by examining all mutations (panels A, D, G), and separately those located in PDZ1 (B, E, H) and PDZ2 (C, F, I).

This analysis revealed minimal correlation between the misfolded ensemble and both the native state and the PDZ1 transition state (Figure 7A–F), suggesting that the misfolded intermediate does not resemble either of these conformations structurally. In particular, mutations within PDZ1 had negligible impact on the stability or population of the misfolded species, reinforcing the idea that PDZ1 remains largely disordered or non‐native within this ensemble. Conversely, a clear and statistically significant correlation was observed when comparing the misfolded trap to the transition state of PDZ2 (Figure 7G–I), especially when considering only mutations located within PDZ2. These results imply that the misfolded intermediate shares meaningful structural similarity with the PDZ2 folding nucleus, while the remainder of the tandem—most notably PDZ1—contributes through nonspecific interactions.

Taken together, these findings suggest a model whereby misfolding arises from the nonspecific collapse of the unfolded PDZ1 domain onto a partially structured PDZ2, particularly during early folding transitions. In this scenario, the PDZ2 transition state nucleates folding, but instead of progressing productively to the native state, the interaction with denatured PDZ1 leads to the formation of a kinetically trapped, misfolded ensemble The misfolded state appears to incorporate some structural elements of the folding nucleus of PDZ2 but arranges them in a frustrated or non‐native topology, such that becomes stabilized by mutations that are otherwise destabilizing to native folding (negative values of Φ). These effects appear to highlight structurally overlapping but energetically antagonistic interactions, consistent with the presence of energetic frustration. Consistently, several mutations that destabilize the native state instead stabilize this misfolded ensemble, yielding negative Φ‐values. These observations highlight structurally overlapping but energetically antagonistic interactions, in line with the presence of energetic frustration. While this description is strongly supported by the mutational dataset, we stress that it represents a working hypothesis; alternative explanations, such as partial domain misregistration or additional non‐native contacts, cannot be excluded.

The interpretation of this misfolded ensemble deserves particular attention in light of prior studies on other PDZ tandems, where transient misfolding has often been attributed to domain swapping.^[^ ^12^, ^25^, ^30^ ^]^ In contrast, the present mutational dataset argues for a distinct mechanism. In fact, while the mutations introduced within PDZ1 do not correspond to a detectable effect on the misfolded trap, several substitutions in PDZ2 have a clear impact on the population and stability of the misfolded ensemble (Table S1, Supporting Information). If the misfolded species arose from a tandem domain‐swapping process, one would expect residues in both PDZ1 and PDZ2 to contribute detectably to the trap. The observed selectivity thus argues strongly against a domain‐swapped origin. Moreover, the correlation analysis between the misfolded ensemble and other states (Figure 7) reinforces this interpretation. Minimal correlation is observed between the misfolded species and either the native state or the PDZ1 transition state, whereas a clear, statistically significant relationship is found with the PDZ2 transition state, particularly when considering only PDZ2 mutations. This indicates that the misfolded ensemble retains meaningful structural similarity to the PDZ2 folding nucleus, while PDZ1 contributes primarily through nonspecific, disordered contacts.

Taken together, these findings support a model in which misfolding arises from the nonspecific collapse of unfolded PDZ1 onto a partially structured PDZ2, yielding a kinetically stabilized but non‐native ensemble that shares features with the PDZ2 transition state. This behavior differs fundamentally from the domain‐swapping mechanisms previously invoked for other systems, as it does not require complementary structural contributions from both domains but rather reflects a transient, intramolecular frustration event specific to the X11 tandem. We speculate that this mechanism may represent an additional general framework by which weak interdomain interactions give rise to folding frustration and kinetic traps in multidomain proteins, as previously observed in other multi‐domain systems.

## 3 Conclusions

We presented here a residue‐level dissection of a misfolded intermediate in a multidomain protein by Φ‐value analysis. While previous studies on other PDZ tandems have invoked transient domain swapping as a source of misfolding,^[^ ^13^, ^18^ ^]^ our data support a distinct mechanism in which non‐native interdomain contacts stabilize a frustrated ensemble that competes with productive folding. This distinction broadens the conceptual framework for understanding how multidomain proteins can transiently misfold, which is to date predominately based on transient domain swapping. Despite the fact that, in the cellular environment, cotranslational folding and chaperone assistance are likely to minimize such misfolding events,^[^ ^60^, ^61^ ^]^ in vitro analyses remain essential to reveal the intrinsic energetic conflicts and frustrated interactions that underlie folding errors.

Through comprehensive Φ‐value analysis and mutational perturbations, we reveal that folding and unfolding follow distinct, asymmetric pathways: unfolding proceeds sequentially, with PDZ2 denaturing before a persistent PDZ1 core, while folding occurs via parallel pathways in which PDZ2 folds rapidly and independently, and PDZ1 folds more slowly, only reaching completion upon autoinhibitory tail binding. This mechanistic asymmetry is rooted in kinetic and structural constraints and highlights the intricate interplay between modular folding and interdomain regulation.

Our results suggest that the transient misfolded trap arises from nonspecific interactions between partially folded PDZ2 and disordered PDZ1, forming a kinetically stable but non‐native ensemble. The structural similarity between this state and the PDZ2 transition state highlights how embryonic folding nuclei may be prone to transient misfolding. Together, these findings reinforce the concept that multidomain folding is governed not only by intrinsic domain properties but also by transient, context‐dependent interactions.

An additional contribution of this work is the direct comparison between folding from free and conformationally restrained denatured ensembles within the same polypeptide. The asymmetry of the X11 tandem provides a natural internal control, allowing us to assess how constraints in the denatured state alter nucleation and pathway selection. This comparison demonstrates that subtle structural biases in the denatured ensemble can reshape folding energetics, offering rare experimental insight into a long‐recognized but difficult‐to‐quantify determinant of protein folding.

Finally, although the experimental approaches used here—stopped‐flow kinetics, Φ‐value analysis, and mutational scanning—are established, their application to a transient misfolded intermediate constitutes a conceptual advance. By extending the scope of Φ‐analysis beyond native transition states, this study provides a framework for dissecting energetic frustration and misfolding in multidomain proteins. We anticipate that similar strategies will be instrumental in uncovering general principles of how multidomain proteins balance productive folding, kinetic traps, and functional adaptability.

## 4 Experimental Section

4.1

4.1.1

##### Protein Mutagenesis, Expression, and Purification

Mutations were designed according to the established rules of Φ‐value analysis,^[^ ^39^, ^41^ ^]^ following the principle of conservative hydrophobic deletion. Standard substitutions include Leu → Ala, Ile → Val, Val → Ala, Thr → Ser, and Ala → Gly replacements at buried positions. These mutations selectively remove native side‐chain interactions while avoiding the introduction of new ones, thereby preserving the integrity of the folding mechanism. All the variants were obtained by site‐directed mutagenesis QuikChange Lightning Mutagenesis Kit (Agilent technologies) following manufacturer instructions. Primers oligos were purchased from Eurofins Genomics and all sequences were confirmed by DNA sequencing.

Proteins were expressed in Escherichia coli BL21 (DE3) (New England Biolabs) cells. Cultures were grown in Luria‐Bertani (LB) medium supplemented with 34 μg mL^−1^ kanamycin at 37 °C. Expression was induced with 1 mM IPTG (isopropyl‐β‐D‐thiogalactopyranoside), followed by overnight incubation at 25 °C. Cells were harvested by centrifugation and resuspended in lysis buffer containing 50 mM Tris‐HCl pH 7.5, 300 mM NaCl, 10 mM imidazole, and protease inhibitor (Complete EDTA‐free, Roche). Cell lysis was performed by sonication, and the clarified lysate was applied to a HisTrap FF column (GE Healthcare) preequilibrated with the same buffer. Proteins were eluted using an imidazole gradient ranging from 10 mM to 1 M. Eluted fractions were buffer‐exchanged into 20 mM Hepes pH 7.5, 300 mM NaCl using a HiTrap Desalting column (GE Healthcare).

##### Kinetic Experiments

Stopped‐flow experiments were performed on an SX‐18 instrument (Applied Photophysics) monitoring changes in intrinsic fluorescence emission. Protein samples were excited at 280 nm, and fluorescence emission was recorded using a 320 nm cut‐off glass filter. The signal thus primarily reports on the emission of the single tryptophan residues located at positions 676 and 767 in PDZ1 and PDZ2, respectively. The protein contains only three tyrosine residues, whose contribution to the total fluorescence is negligible, being approximately tenfold weaker than that of tryptophan. Notably, a tryptophan residue was engineered in PDZ2 to allow its selective detection in the tandem construct, as described in ref. [17]. At least five individual traces were acquired and then averaged for each experiment, and all averaged traces were satisfactorily fitted with a double exponential equation. Experiments were conducted using a typical protein concentration of 2 μM (after mixing) in 50 mM Hepes pH 7.5 at 37 °C and varying urea concentrations. Double jump experiments were performed in analogy to what described earlier.^[^ ^24^ ^]^ In particular, all the variants were subjected to a sequential mixing double‐jump experiment involving interrupted unfolding. The experiment was design to (i) first accumulate the intermediate displaying a denatured PDZ2 and a native PDZ1 with the bound tail and (ii) interrupt the unfolding by mixing with refolding buffer to monitor the folding rate constant of PDZ2 with a bound tail. In practice, the proteins in 2M urea were first mixed with 8M urea to obtain a final concentration of 5M urea, then, after a controlled delay time, a second mix was performed to achieve refolding at different final urea concentrations. All data were consistent with a single exponential behavior and were added do the chevron plots reported in Figure 2.

Data analysis was performed as extensively discussed in ref. [24]. In particular, for each variant, the kinetic chevron plots were globally fitted to the system of equations shown in Figure 1, which describes the minimal folding mechanism of X11 PDZ1–PDZ2. To simplify the analysis and increase the robustness of the estimated rate constants, the kinetic *m*‐values were shared across all datasets. This assumption, commonly used in Φ‐value analysis,^[^ ^39^, ^40^, ^41^ ^]^ implies that the overall change in solvent‐accessible surface area along the reaction coordinate is minimally perturbed by the conservative mutations introduced and is supported by the mutational design strategy adopted here. From the fitted microscopic rate constants, Φ‐values were then calculated for each individual step, as defined by the states in Figure 1, thereby allowing residue‐level structural information to be obtained for the productive and nonproductive intermediates. The obtained values are reported in Table 1. In practice, the mutation‐induced free energy changes of a putative *i* state were derived as

(1) $$\Delta \Delta G_{D-i}=RT\cdot \text{ln}\frac{k_{D-i}^{\text{wt}}}{k_{D-i}^{\text{mut}}}$$

Finally, to calculate the Φ values, the following equations were applied

(2) $$\Delta \Delta G_{D-N}=RT\cdot \text{ln}\frac{k_{D-N}^{\text{wt}}}{k_{D-N}^{\text{mut}}}$$

(3) $$\Phi_{i}=\frac{\Delta \Delta G_{D-i}}{\Delta \Delta G_{D-N}}$$

The values reported in Table 1 refer to the unfolding and folding process as probed by Φ value analysis. Hence to calculate the values, we used the following equations: for the unfolding Φ values

(4) $$\Phi_{D-1}=1-\;\frac{\Delta \Delta G_{N-1}}{\Delta \Delta G_{D-N}}\text{ where }\Delta \Delta G_{N-1}=RT\cdot \text{ln}\frac{k_{U}^{\text{PDZ}2\text{ bound wt}}}{k_{U}^{\text{PDZ}2\text{ bound mut}}}$$

(5) $$\Phi_{D-2}=1-\frac{\Delta \Delta G_{N-2}}{\Delta \Delta G_{D-N}}\text{ where }\Delta \Delta G_{N-2}=RT\cdot \left(\text{ln}\frac{k_{F}^{\text{PDZ}2\text{ bound wt}}}{k_{U}^{\text{PDZ}2\text{ bound wt}}}\;-\text{ ln}\frac{k_{F}^{\text{PDZ}2\text{ bound mut}}}{k_{U}^{\text{PDZ}2\text{ bound mut}}}\;\right)$$

(6) $$\Phi_{D-3}=1-\;\frac{\Delta \Delta G_{N-3}}{\Delta \Delta G_{D-N}}\text{ where }\Delta \Delta G_{N-3}=RT\cdot \text{ln}\frac{k_{U}^{\text{PDZ}1-\text{PDZ}2\text{* wt}}}{k_{U}^{\text{PDZ}1-\text{PDZ}2\text{* mut}}}\;$$

for the refolding Φ values

(7) $$\Phi_{D-5}=1-\;\frac{\Delta \Delta G_{D-5}}{\Delta \Delta G_{D-N}}\text{ where }\Delta \Delta G_{D-5}=RT\cdot \text{ln}\frac{k_{F}^{\text{PDZ}2\text{ free wt}}}{k_{F}^{\text{PDZ}2\text{ free mut}}}$$

(8) $$\Phi_{D-6}=1-\frac{\Delta \Delta G_{D-6}}{\Delta \Delta G_{D-N}}\text{ where }\Delta \Delta G_{D-6}=RT\cdot \left(\text{ln}\frac{k_{F}^{\text{PDZ}2\text{ free wt}}}{k_{U}^{\text{PDZ}2\text{ free wt}}}\;-\text{ ln}\frac{k_{F}^{\text{PDZ}2\text{ free mut}}}{k_{U}^{\text{PDZ}2\text{ free mut}}}\;\right)$$

(9) $$\Phi_{D-7}=1-\;\frac{\Delta \Delta G_{D-7}}{\Delta \Delta G_{D-N}}\text{ where }\Delta \Delta G_{D-7}^{2002}=RT\cdot \text{ln}\frac{k_{F}^{\text{PDZ}1\text{ wt}}}{k_{F}^{\text{PDZ}1\text{ mut}}}\;$$

where the number associated to each state is reported in Figure 3.

## Supporting Information

Supporting Information is available from the Wiley Online Library or from the author.

## Conflict of Interest

The authors declare no conflict of interest.

## Supporting information

Supplementary Material

## Data Availability

The data that support the findings of this study are available from the corresponding author upon reasonable request.
