# Supplementary material for: Dissecting the Structural Features of Folding Asymmetry and Transient Misfolding in a Multidomain PDZ Scaffold
Source: Small Sci. 2025 Dec 10;6(1):e202500511. doi: 10.1002/smsc.202500511 (PMC12798790; doi:10.1002/smsc.202500511)
Supplement: Supplementary file 1 — Supplementary Material [file SMSC-6-e202500511-s001.pdf]

SUPPORTING INFORMATION

Dissecting the structural features of folding asymmetry and transient misfolding in a Multidomain PDZ Scaffold

Valeria Pennacchietti[1], Mariana Di Felice[1], Julian Toso[1], Lucia Marcocci[1], Paola Pietrangeli[1], Eduarda S. Ventura[1], Francesca Malagrinò[2], Angelo Toto[1] and Stefano Gianni[1,*]

[1]Dipartimento di Scienze Biochimiche "A. Rossi Fanelli", Sapienza Università di Roma, P.le Aldo Moro 5, 00185, Rome, Italy – Laboratory affiliated to Istituto Pasteur Italia - Fondazione Cenci Bolognetti

[3] Dipartimento di Medicina clinica, sanità pubblica, scienze della vita e dell'ambiente, Università dell'Aquila, Piazzale Salvatore Tommasi 1, 67010 L'Aquila – Coppito, Italy

*Corresponding Author: Stefano Gianni (stefano.gianni@uniroma1)

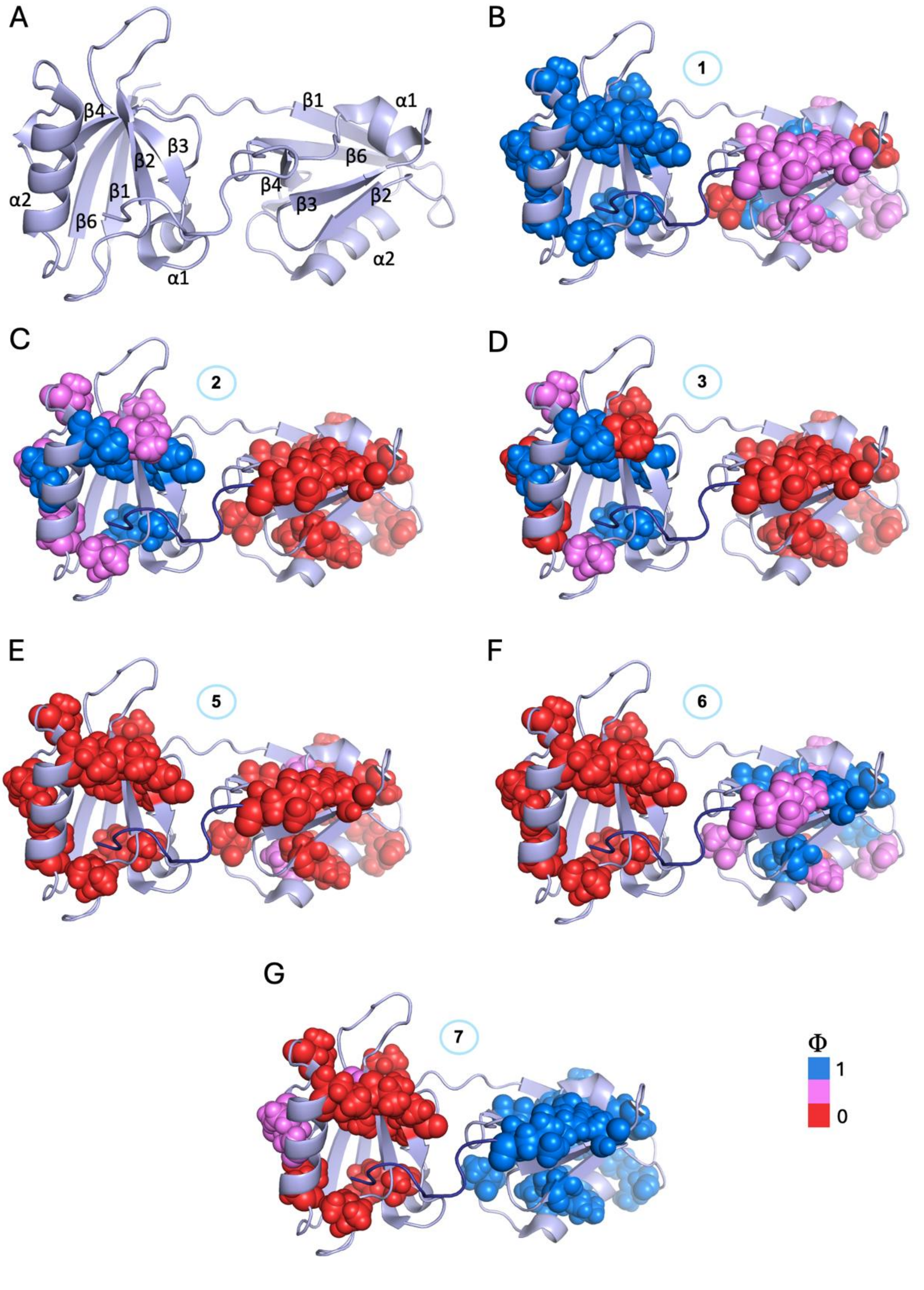

Figure S1. Structural distribution of measured Φ values for the different states depicted in Figure 3.

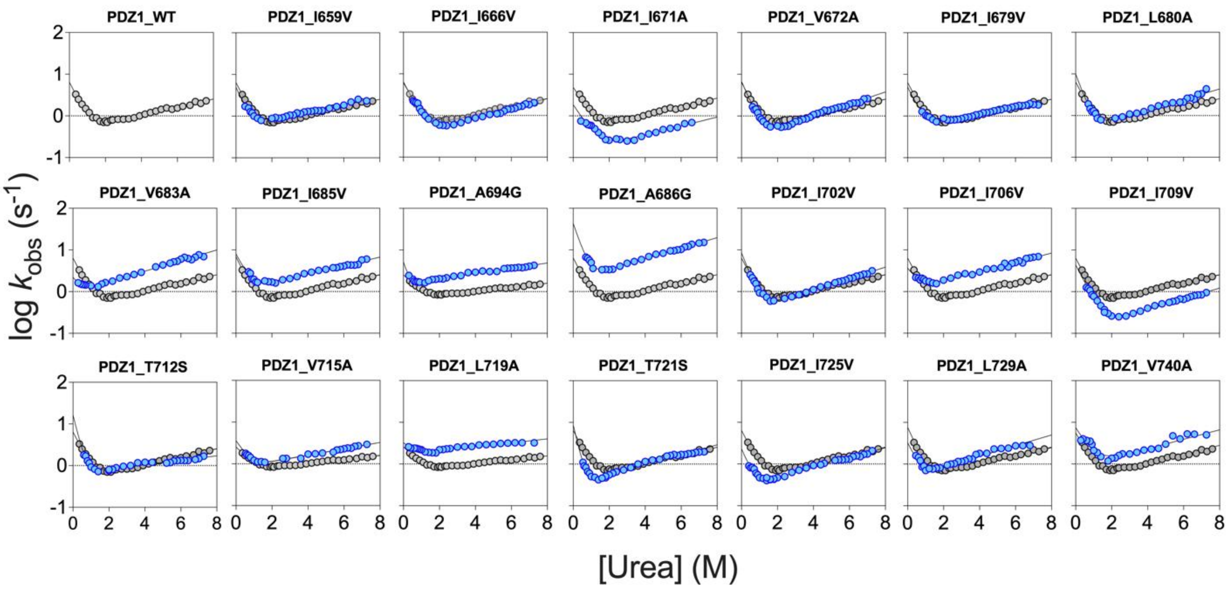

Figure S2. Chevron plots of PDZ1 in isolation and its variants. Lines are the best fit to a two state model.

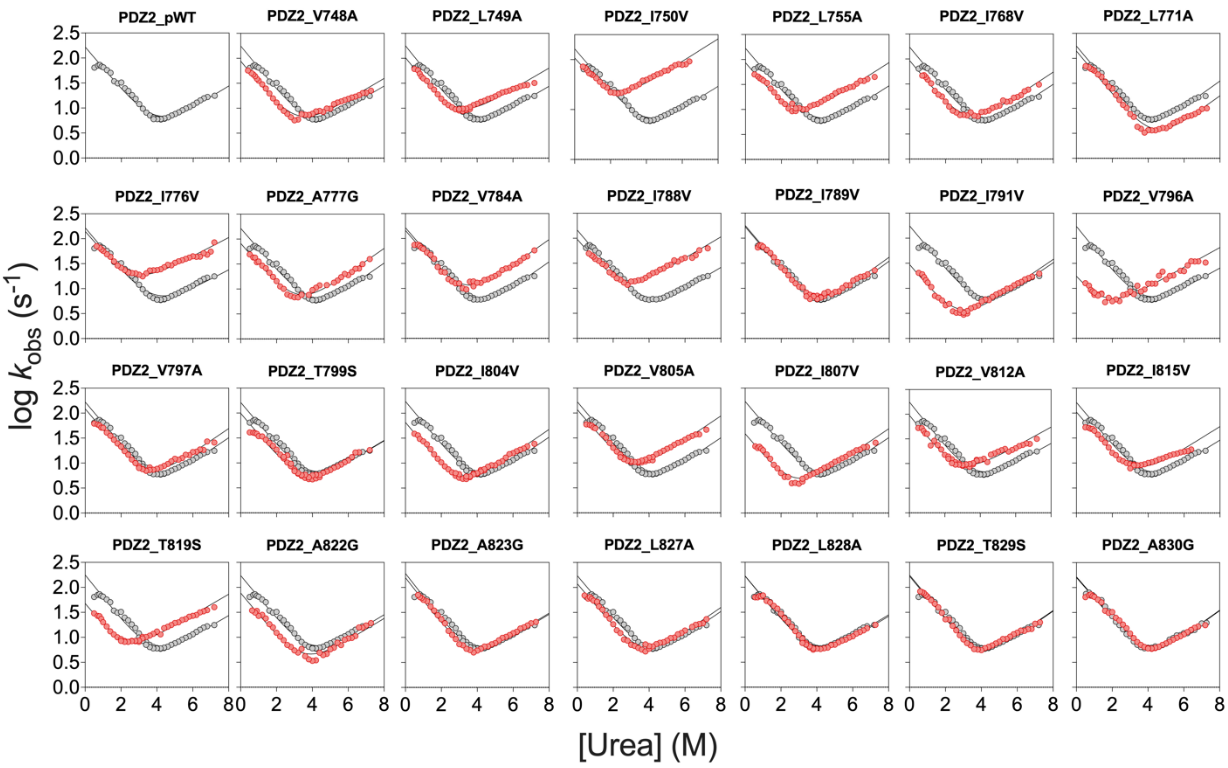

Figure S3. Chevron plots of PDZ2 in isolation and its variants. Lines are the best fit to a two state model.

Table S1. Thermodynamic parameters of PDZ1-DZ2 variants.

	$\Delta\Delta G_{N-1}$ (kcal mol^{-1})	$\Delta\Delta G_{N-2}$ (kcal mol^{-1})	$\Delta\Delta G_{N-3}$ (kcal mol^{-1})	$\Delta\Delta G_{D-5}$ (kcal mol^{-1})	$\Delta\Delta G_{D-6}$ (kcal mol^{-1})	$\Delta\Delta G_{D-7}$ (kcal mol^{-1})	$\Delta\Delta G_{mis}$ (kcal mol^{-1})
**I659V**	-0.03 ± 0.05	0.020 ± 0.03	0.34 ± 0.02	0	-0.030 ± 0.01	0.12 ± 0.01	0.11 ± 0.01
**I666V**	-0.04 ± 0.05	0.33 ± 0.02	0.40 ± 0.03	-0.06 ± 0.02	-0.05 ± 0.02	-0.16 ± 0.01	0.060 ± 0.01
**I671V**	0.06 ± 0.05	0.40 ± 0.02	-0.07 ± 0.01	-0.010 ± 0.05	0.05 ± 0.03	0.070 ± 0.02	-0.18 ± 0.01
**V672A**	-0.03 ± 0.03	0.32 ± 0.03	0.17 ± 0.01	-0.02 ± 0.002	-0.05 ± 0.01	0.050 ± 0.03	0.20 ± 0.01
**I679V**	-0.07 ± 0.05	-0.06 ± 0.04	0.09 ± 0.01	0.01 ± 0.03	-0.06 ± 0.02	0.050 ± 0.02	0.23 ± 0.02
**L680A**	0.01 ± 0.01	0.21 ± 0.02	0.07 ± 0.01	-0.01 ± 0.02	-0.07 ± 0.03	0.050 ± 0.01	0.003 ± 0.01
**V683A**	-0.19 ± 0.07	-0.27 ± 0.04	0.88 ± 0.03	0.03 ± 0.02	-0.16 ± 0.01	0.43 ± 0.01	0.080 ± 0.02
**I685V**	-0.15 ± 0.04	-0.19 ± 0.05	0.85 ± 0.05	-0.06 ± 0.02	-0.15 ± 0.01	0.050 ± 0.02	-0.010 ± 0.01
**A686G**	-0.54 ± 0.07	-0.48 ± 0.02	1.05 ± 0.10	-0.13 ± 0.04	-0.67 ± 0.02	-0.06 ± 0.02	-0.040 ± 0.02
**A694G**	-0.03 ± 0.05	0.07 ± 0.02	2.17 ± 0.20	-0.01 ± 0.03	-0.04 ± 0.01	-0.18 ± 0.01	-0.34 ± 0.02
**I702V**	-0.021 ± 0.04	0.20 ± 0.04	0.14 ± 0.02	0.022 ± 0.02	0.005 ± 0.05	0.060 ± 0.01	0.06 ± 0.01
**I706V**	-0.05 ± 0.06	0.01 ± 0.002	0.76 ± 0.04	0.11 ± 0.03	0.060 ± 0.01	0.14 ± 0.05	0.22 ± 0.02
**I709V**	0.05 ± 0.05	0.26 ± 0.08	0.070 ± 0.03	-0.01 ± 0.03	0.05 ± 0.03	0.070 ± 0.05	0.18 ± 0.01
**T712S**	0.05 ± 0.07	0.33 ± 0.03	0.020 ± 0.01	-0.040 ± 0.02	0.05 ± 0.02	0.48 ± 0.02	0.05 ± 0.02
**V715A**	-0.06 ± 0.08	0.32 ± 0.02	0.46 ± 0.03	0.14 ± 0.02	0.08 ± 0.02	0.020 ± 0.08	0.24 ± 0.01
**L719A**	-0.33 ± 0.04	-0.06 ± 0.04	1.25 ± 0.08	0.02 ± 0.01	-0.31 ± 0.02	0.06 ± 0.04	-0.18 ± 0.06
**T721S**	-0.73 ± 0.06	-0.47 ± 0.02	0.26 ± 0.02	0.13 ± 0.01	-0.60 ± 0.02	-0.05 ± 0.05	0.28 ± 0.05
**I725V**	-0.06 ± 0.03	0.03 ± 0.06	0.18 ± 0.01	0.08 ± 0.01	0.02 ± 0.001	0.34 ± 0.01	0.22 ± 0.01

**L729A**	0.04 ± 0.03	0.39 ± 0.02	0.12 ± 0.01	0.090± 0.02	0.13 ± 0.03	0.30 ± 0.02	0.07 ± 0.02
**V740A**	0.07 ± 0.03	0.53 ± 0.04	0.23 ± 0.01	-0.10 ± 0.01	-0.030 ± 0.02	0.16 ± 0.01	0.06 ± 0.04
**V748A**	0.25 ± 0.05	1.05 ± 0.10	-0.090 ± 0.02	0.54 ± 0.02	0.80 ± 0.04	0.060 ± 0.001	0.003 ± 0.002
**L749A**	0.44 ± 0.07	1.05 ± 0.06	-0.080 ± 0.02	0.23 ± 0.02	0.68 ± 0.02	0.45 ± 0.01	-0.10 ± 0.01
**I750V**	0.79 ± 0.04	1.34 ± 0.07	-0.16 ± 0.01	0.16 ± 0.01	0.95 ± 0.02	0.08 ± 0.01	0.10 ± 0.02
**L755A**	0.43 ± 0.04	0.69 ± 0.03	-0.01 ± 0.05	-0.06 ± 0.04	0.43 ± 0.04	-0.003 ± 0.01	-0.20 ± 0.01
**V763A**	0.84 ± 0.05	1.33 ± 0.14	-0.09 ± 0.08	0.70 ± 0.04	1.54 ± 0.10	0.65 ± 0.02	0.21 ± 0.01
**I768V**	0.24 ± 0.05	0.59 ± 0.03	-0.01 ± 0.01	0.13 ± 0.02	0.37 ± 0.04	-0.08 ± 0.02	-0.25 ± 0.02
**L771A**	1.02 ± 0.06	1.70 ± 0.10	-0.01 ± 0.01	0.46 ± 0.08	1.48 ± 0.10	0.19 ± 0.01	0.27 ± 0.01
**I776V**	0.31 ± 0.02	0.43 ± 0.01	0.01 ± 0.02	0.05 ± 0.05	0.36 ± 0.04	-0.03 ± 0.03	0.090 ± 0.01
**A777G**	0.26 ± 0.04	0.45 ± 0.01	0.02 ± 0.02	-0.34 ± 0.06	-0.08 ± 0.03	-0.02 ± 0.01	-0.07 ± 0.02
**V782A**	0.28 ± 0.04	0.66 ± 0.03	-0.16 ± 0.01	-0.33 ± 0.01	-0.05 ± 0.03	0.030 ± 0.01	-0.78 ± 0.01
**V784A**	0.93 ± 0.05	1.61 ± 0.10	-0.12 ± 0.01	0.13 ± 0.02	1.06 ± 0.10	0.12 ± 0.01	0.09 ± 0.02
**I788V**	0.30 ± 0.06	1.04 ± 0.10	-0.18 ± 0.02	0.24 ± 0.04	0.54 ± 0.02	0.28 ± 0.02	-0.55 ± 0.01
**I789V**	0.11 ± 0.05	0.45± 0.03	-0.060 ± 0.03	0.07 ± 0.02	0.19 ± 0.01	-0.11 ± 0.01	0.23 ± 0.02
**I791V**	0.25 ± 0.06	1.25 ± 0.10	-0.36 ± 0.02	0.67 ± 0.03	0.93 ± 0.03	0.030 ± 0.01	-0.16 ± 0.01
**V796A**	0.61 ± 0.04	2.7± 0.20	-1.36 ± 0.10	0.77 ± 0.02	1.38 ± 0.10	0.63 ± 0.02	-0.16 ± 0.01
**V797A**	0.52 ± 0.02	0.60 ± 0.03	0.02 ± 0.01	-0.11 ± 0.01	0.40 ± 0.02	0.14 ± 0.01	-0.48 ± 0.02
**T799S**	-0.19 ± 0.04	0.31 ± 0.02	-0.14 ± 0.01	0.37 ± 0.01	0.17 ± 0.01	0.06 ± 0.01	0.27 ± 0.01
**I804V**	0.40 ± 0.02	1.14 ± 0.10	-0.13 ± 0.02	0.77 ± 0.03	1.17 ± 0.10	0.04 ± 0.02	0.69 ± 0.03
**V805A**	0.55 ± 0.06	1.00 ± 0.06	-0.11 ± 0.01	0.17 ± 0.01	0.72 ± 0.04	0.66 ± 0.01	-0.04 ± 0.01
**I807V**	0.25 ± 0.07	1.40 ± 0.10	0.080 ± 0.05	-0.23 ± 0.01	0.02 ± 0.01	0.04 ± 0.02	-0.74 ± 0.02

**V812A**	0.52 ± 0.07	1.12 ± 0.10	0.01 ± 0.01	0.35 ± 0.02	0.87 ± 0.05	-0.002 ± 0.02	0.27 ± 0.01
**I815V**	0.44 ± 0.05	1.07 ± 0.05	-0.13 ± 0.02	0.42 ± 0.03	0.86 ± 0.04	0.007 ± 0.001	0.10 ± 0.01
**T819S**	0.73 ± 0.04	1.50 ± 0.08	-0.15 ± 0.01	0.59 ± 0.01	1.32 ± 0.10	0.42 ± 0.02	0.09 ± 0.04
**A822G**	-0.18 ± 0.06	0.32 ± 0.02	0.060 ± 0.04	0.35 ± 0.02	0.16 ± 0.01	-0.18 ± 0.01	0.27 ± 0.01
**A823G**	-0.09 ± 0.02	0.21 ± 0.01	-0.61 ± 0.03	0.02 ± 0.02	-0.06 ± 0.03	0.69 ± 0.02	-0.43 ± 0.01
**L827A**	-0.43 ± 0.05	-0.21 ± 0.01	0.07 ± 0.01	0.1 ± 0.06	-0.33 ± 0.01	0.12 ± 0.01	0.32 ± 0.02
**L828A**	0.26 ± 0.07	0.80 ± 0.04	-0.02 ± 0.03	0.13 ± 0.01	0.39 ± 0.01	-0.17 ± 0.01	-0.12 ± 0.01
**T829S**	0.08 ± 0.08	0.25 ± 0.01	0.11 ± 0.01	-0.090 ± 0.03	-0.010 ± 0.02	0.08 ± 0.03	-0.31 ± 0.02
**A830G**	0.06 ± 0.02	0.21 ± 0.01	-0.16 ± 0.01	-0.080 ± 0.04	-0.020 ± 0.001	-0.050 ± 0.01	-0.55 ± 0.01

By following standard rules of Φ values, mutations leading a change in stability of less than 0.4 kcal mol^{-1} were excluded from the analysis.

Table S2. Thermodynamic parameters of PDZ1 and PDZ2 variants in isolation.

	$\Delta\Delta G_{TS}$ (kcal mol^{-1})	$\Delta\Delta G_{D-N}$ (kcal mol^{-1})	ϕ
**PDZ1**			
I659V	0.26 ± 0.03	0.40 ± 0.02	0.68 ± 0.05
I666V	-0.06 ± 0.01	-0.17 ± 0.03	-
I671V	0.80 ±0.01	0.13 ± 0.05	-
V672A	0.24 ± 0.02	0.24 ± 0.07	-
I679V	0.12 ± 0.03	0.16 ± 0.02	-
L680A	0.18 ± 0.05	0.41 ± 0.03	0.43 ± 0.03
V683A	1.40 ± 0.10	2.10 ± 0.20	0.67 ± 0.03
I685V	-0.10 ± 0.04	0.46 ± 0.02	-0.21 ± 0.02
A686G	-0.60 ± 0.05	0.50 ± 0.01	-1.18 ± 0.01
A694G	0.40 ± 0.03	1.60 ± 0.05	0.24 ± 0.03
I702V	0.15 ± 0.05	0.21 ± 0.07	-
I706V	0.45 ± 0.02	1.11 ± 0.05	0.41 ± 0.01
I709V	0.20 ± 0.06	-0.35 ± 0.05	-0.57 ± 0.02
T712S	0.14 ± 0.07	0.10 ± 0.05	-
V715A	-0.05 ± 0.03	0.61 ± 0.03	-0.10 ± 0.01
L719A	-0.05 ± 0.02	1.10 ± 0.10	-0.04 ± 0.02
T721S	0.90 ± 0.08	0.84 ± 0.02	1.04 ± 0.20
I725V	0.95 ± 0.05	0.82 ± 0.06	1.16 ± 0.10
L729A	0.55 ±0.02	0.80 ± 0.06	0.69 ± 0.05
V740A	0.07 ± 0.06	3.21 ± 0.2	0.02 ± 0.01
**PDZ2**			
V748A	0.45 ± 0.03	0.66 ± 0.04	0.69 ± 0.03
L749A	0.36 ± 0.02	0.86 ± 0.07	0.42 ± 0.01
I750V	0.30 ± 0.02	1.65 ± 0.10	0.22 ± 0.02
L755A	0.46 ± 0.01	1.00 ± 0.10	0.43 ± 0.01
V763A	-	-	-
I768V	0.42 ± 0.03	0.73 ± 0.05	0.57 ± 0.03
L771A	0.60 ± 0.02	0.42 ± 0.01	1.39 ± 0.05
I776V	0.10 ± 0.02	0.97 ± 0.02	0.10 ± 0.01
A777G	0.46 ± 0.04	0.86 ± 0.04	0.54 ± 0.03
V782A	-	-	-

V784A	0.20 ± 0.01	0.84 ± 0.10	0.24 ± 0.01
I788V	0.50 ± 0.04	1.34 ± 0.05	0.36 ± 0.03
I789V	0.09 ± 0.03	0.24 ± 0.02	-
I791V	1.14 ± 0.10	1.18 ± 0.07	0.97 ± 0.03
V796A	1.45 ± 0.10	1.97 ± 0.10	0.74 ± 0.04
V797A	0.22 ± 0.01	0.46 ± 0.02	0.48 ± 0.02
T799S	0.32 ± 0.03	0.35 ± 0.03	-
I804V	0.66 ± 0.03	0.83 ± 0.10	0.79 ± 0.06
V805A	0.28 ± 0.01	0.84 ± 0.08	0.33 ± 0.01
I807V	0.97 ± 0.05	1.17 ± 0.10	0.83 ± 0.02
V812A	0.46 ± 0.02	0.93 ± 0.10	0.5 ± 0.01
I815V	0.30 ± 0.01	0.62 ± 0.03	0.49 ± 0.02
T819S	0.87 ± 0.04	1.44 ± 0.08	0.6 ± 0.01
A822G	0.60 ± 0.02	0.54 ± 0.02	1.1 ± 0.10
A823G	0.12 ± 0.03	0.15 ± 0.01	-
L827A	-	-	-
L828A	0.08 ± 0.02	-0.03 ± 0.01	-
T829S	0.04 ± 0.02	0.05 ± 0.03	-
A830G	0.01 ± 0.04	0.03 ± 0.01	-

Φ values were calculated assuming a simple two state model. By following standard rules of Φ values, mutations leading a change in stability of less than 0.4 kcal mol^{-1} were excluded from the analysis. The mutants V763A, V782A and L827A could not be expressed successfully.
